# Some Properties of Densified Eastern Redcedar as Function of Heat and Pressure

**DOI:** 10.3390/ma10111275

**Published:** 2017-11-07

**Authors:** Onur Ulker, Salim Hiziroglu

**Affiliations:** 1 Department of Interior Architecture and Environmental Design, University of Kırıkkale, Yahşihan 71450, Turkey; 2 Department of Natural Resource Ecology and Management, Oklahoma State University, Stillwater, OK 74078-6013, USA; salim.hiziroglu@okstate.edu

**Keywords:** eastern redcedar, compression, heat treatment, roughness, hardness, color change

## Abstract

The objective of this study was to evaluate some of the properties of densified eastern redcedar as function of temperature and pressure. Surface quality, adhesive bondline shear strength, hardness, and color changes of the samples compressed using different temperature levels ranging from 100 °C to 180 °C were investigated. Based on the findings in this work, surface roughness of compressed specimens decreased with increased temperature. Overall adhesive bondline shear strength of the samples decreased as compared to that of control specimens as a result of compression. It appears that densified samples exposed to a temperature of 180 °C had significantly darker surface than those of the others, based on color measurement. Data found in this work provide some basic information for more efficient use of underutilized species such as eastern redcedar.

## 1. Introduction

Densification of wood has been used for many years for different applications [1,2]. The typical densification process of wood is defined as compression by pressing with a combination of heat treatment or filling the cells with the application of different materials, including polymer or natural resins [3,4,5,6]. When heat treatment is combined with compression, certain properties of the wood such as surface roughness, color modification, and dimensional stability can be enhanced. One of the most important advantages of wood densification is to improve major mechanical properties of the member, and this approach plays a more important role in the case of the densification of low-density species. The thermomechanical compression and densification of wood generally take place in four distinctive stages. These are softening, plastic softening, and plasticizing of the cell wall compression in a radial grain orientation, climatizing in a densified unit, and fixation of dimensional stability [5,7,8,9].

Species, temperature level, softening time span, pressure, and densification method are major parameters influencing the final properties of densified material [10]. In a previous study, the surface roughness of compressed birch and alder veneer samples was evaluated [11]. It was found that overall surface quality of the densified samples increased with compression level [11]. Surface quality of thermally compressed Douglas fir veneer samples was also investigated in a past study [12]. Based on roughness measurements carried out using a stylus-type equipment, specimens compressed with higher pressure and temperature had smoother surface than those exposed to lower pressure and temperature levels.

Aydin and Colakoglu evaluated the influence of veneer drying temperature on surface roughness of plywood panels, and it was concluded that samples from alder and spruce species had enhanced surface quality with increasing temperature [13]. It was also evaluated of bonding strength of heat treated and compressed Eastern redcedar and it was found that combination of heat treatment and compression enhanced overall surface quality of the samples in the form of their roughness determined using a stylus type equipment [14]. Hydrothermal treatment of wood samples in a closed chamber was employed in a past study [1]. In this approach, the wood specimens were compressed by employing a heated setup in one step before they were cooled off. This idea is relatively related to the Staypak method [15]. Surface densification of maple specimens was also carried out using heat and compression in a continuous belt type press by heating up the surface of the sample to a temperature of 220 °C [16].

Eastern redcedar (*Juniperus virginiana* L.) is one of the widely distributed species in Oklahoma. The current area covered by eastern redcedar in Oklahoma is estimated to be beyond 4.5 million hectares, and it was projected to be 6.3 million ha by 2013 [17]. The cedar population is growing at the rate of 380 hectares per day, resulting in a significant adverse impact on ecology. The cost of problem caused by eastern redcedar invasion was approximately $447 million in 2013 [17]. Although properties of eastern redcedar have been comprehensively investigated and evaluated, there is very little or no information on surface properties, hardness, and color changes of such species as function of thermal compression process. Therefore, the objective of this study was to determine such properties of eastern redcedar samples compressed in a hot press. Data from this work could be beneficial for more efficient utilization of this under-utilized species for value-added products with enhanced properties. 

## 2. Materials and Methods

### 2.1. Preparation of Samples and Applied Tests

**Figure 1:**
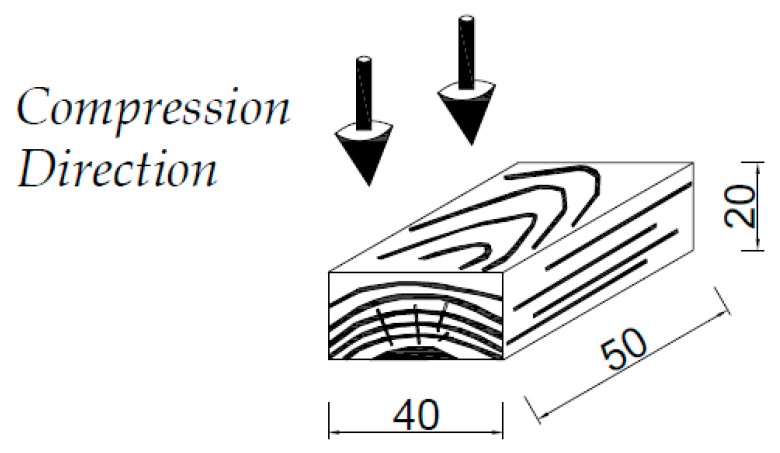
Compression direction and sample size (mm) before densification.

Defect-free eastern redcedar (*Juniperus virginiana* L.) strips with dimension of 5 cm by 4 cm by 2 cm were supplied by a local sawmill in Oklahoma, USA. After this they were kept in a conditioned chamber with a temperature of 20 °C and relative humidity of 65% until they reached equilibrium moisture content of 12%. A total of 175 samples with 2.5 m length were cut from long strips with tangential grain orientation, as illustrated in Figure 1. 

Tangential surfaces of each sample were sanded sequentially with 100 and 200 grit sand paper with several light strokes. A total of 125 samples—25 for each temperature level (100 °C, 120 °C, 150 °C, 180 °C) and control sample—were compressed on their tangential surface on a Carver press with a capacity of 10 ton. Samples were compressed using a pressure of 6.08 MPa at each temperature level for 20 min. Both control and compressed samples were kept in the conditioning room for several days prior to any tests. Table 1 displays sample size and testing schedule.

### 2.2. Moisture Content and Density of the Samples

Moisture content of the control samples was determined by measuring their initial and oven-dried weight at an accuracy of 0.01 g after they were kept in an oven at a temperature of 103 ± 2 °C. The following equation was employed for moisture content calculation of the samples. Samples were taken from the oven and kept in a dry chamber. Moisture content was calculated with this formula:

Moisture Content (%) = [(Mass When Cut − Oven Dry Mass)/Oven Dry Mass] × 100%

Density of each samples was also determined by weighting and measuring dimensions at accuracy levels of 0.01 g and 0.1 cm, respectively.

### 2.3. Densification Process

Samples were cut from the strips to the dimensions of 50 mm by 40 mm by 20 mm (length × width × thickness). In the next step, the samples were stacked and left to dry naturally according to standards. They were kept in a climate chamber at a temperature of 20 ± 2 °C and a relative humidity of 65 ± 5% until they reached constant weight.

The press temperature was adjusted to the target temperature levels at an accuracy of ±1 °C. The samples were placed on the lower platen in such a way that the pressure would be applied in a radial direction. In order to ensure heat transfer to both sides, the upper flat surface was contacted on surfaces without applying pressure. The samples were kept until they reached the target temperature values of 100 °C 120 °C, 150 °C, and 180 °C. 

**Figure 2:**
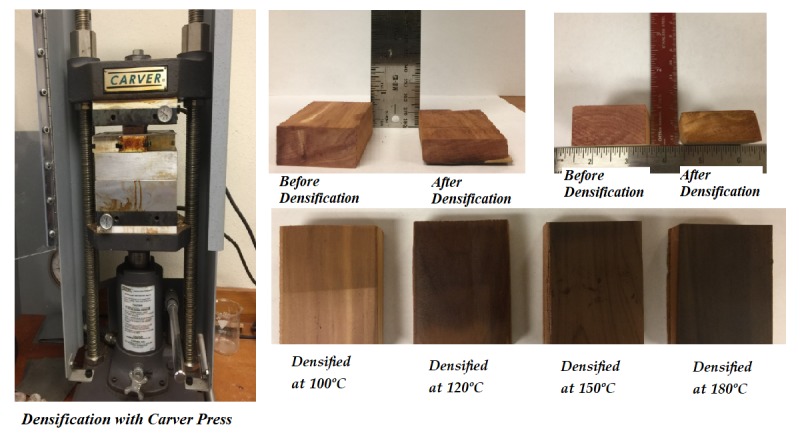
Densification process with Carver laboratory press and samples after densification.

**Figure 3:**
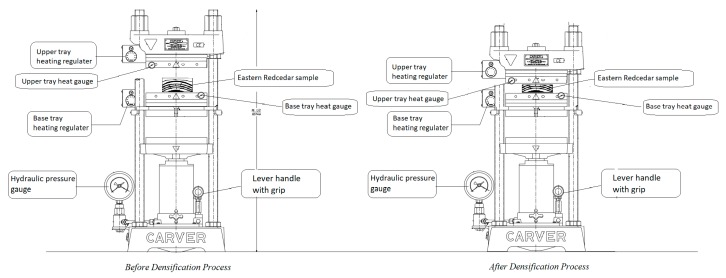
Densification process of the samples in Carver laboratory press.

The pressure gauge of the press was adjusted to provide a densification pressure of 6.08 MPa. The samples were densified by being compressed manually with a 20 m/min loading speed, as shown in Figure 2. Samples were pressed at constant pressure to eliminate possible spring-back effect, as illustrated in Figure 3. In the next step, samples were removed from the press and kept in room condition for 7 days. 

### 2.4. Surface Roughness Measurement

**Figure 4:**
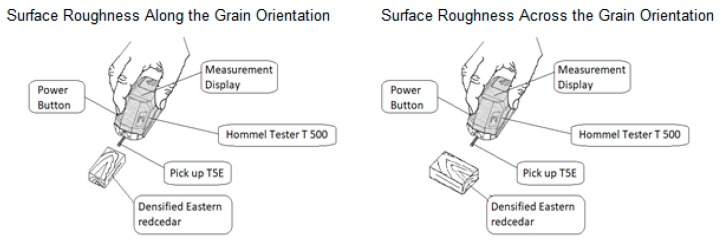
Surface roughness measurement test.

A stylus type T-500 Hommel America portable profilometer (Hommel Inc., Detroit, MI, USA) was employed for surface roughness measurement. Six measurements with a tracing span of 15.2 mm were taken from both sides of the samples across and along the grain orientation by employing a Hommel T-500 profilometer before and after heat treatment and compression of the samples. The stylus unit used in this study consists of the main unit and the pickup model TkE. The pickup has a skid type diamond stylus with 5 μm tip radius and a 90-tip angle. The stylus traverses the surface at constant speed of 1 mm/s over 15.2 mm tracing length, converting the vertical displacement of the stylus into an electrical signal [18]. The calibration of the instrument was checked every 100 measurements by using a standard reference plate with Ra values of 3.02 μm and 0.48 μm. A cut-off length of 2.54 mm—a parameter that differentiates roughness and waviness profiles from each other—was used for the test as illustrated in Figure 4 [18].

### 2.5. Hardness Measurement of the Samples 

**Figure 5:**
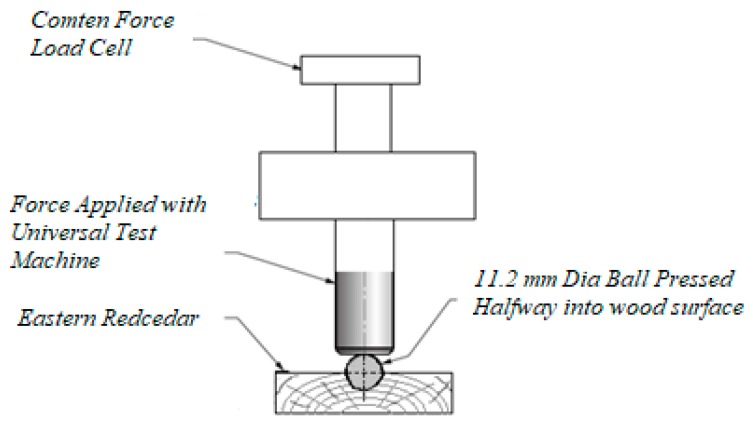
Janka hardness measurement with Comten 95 Series Universal Testing machine.

Hardness of the control and treated specimens was tested by embedding a hemisphere steel having 11.2 mm diameter onto their tangential surface to the grain directions using a Comten 95 Series Universal Testing machine (Com-Ten Industries, Pinellas Park, FL, USA). Four measurements were taken from each sample and recorded in lbs to evaluate their Janka hardness, as illustrated in Figure 5 [19].

### 2.6.Adhesive Bondline Shear Strength Process

**Figure 6:**
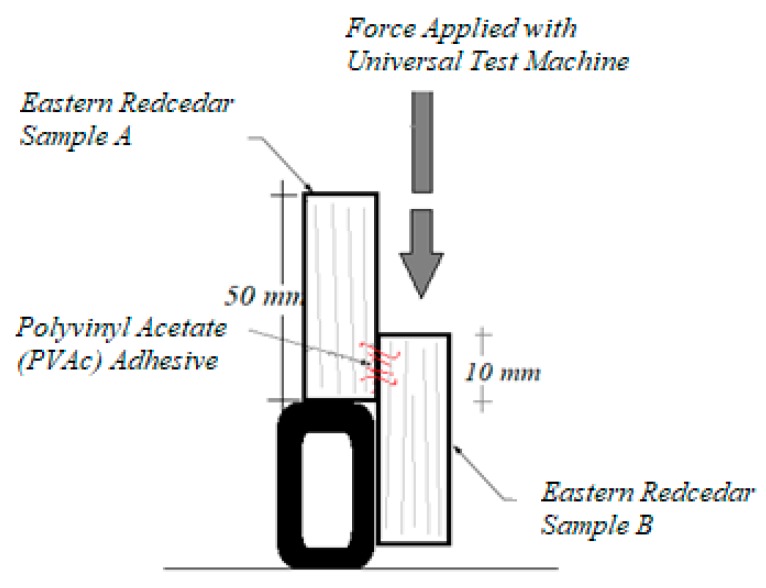
Adhesive bondline shear strength measurement with Comten 95 Series Universal Testing machine.

Polyvinyl acetate (PVAc) adhesive was used to bond samples in the form of pair to determine their adhesive bondline shear strength. Adhesive was applied to both surfaces of each bondline shear pair at a spread rate of 120 g/m². The pair was then cold pressed using an approximate pressure of 40.8 kg/cm^2^ for 20 min at room temperature before bondline shear tests were carried out. Adhesive bondline shear strength test was also carried out on a Comten testing unit (Com-Ten Industries, Pinellas Park, FL, USA) equipped with a load cell having capacity of 1000 kg, as illustrated in Figure 6 [17]. 

### 2.7. Color Measurement of the Samples

The color measurement of all specimens was recorded on the surface of specimens before and after heat treatment with a colorimeter FRU WR-10QC (Shenzhen Wave Optoelectronics Technology Co Ltd, Shenzhen, China). The sensor head was 8 mm in diameter. Measurements were carried out using a D65 illuminant and 10 degree standard observer (Shenzhen Wave Optoelectronics Technology Co Ltd., Shenzhen, China). Percentage of reflectance, collected at 10 nm intervals over the visible spectrum ranging from 400 to 700 nm, was converted into the CIELAB color system (Shenzhen Wave Optoelectronics Co Ltd., Shenzhen, China), where L* describes the lightness, and a* and b* describe the chromatic coordinates on the green-red and blue-yellow axes, respectively. 

From the L*, a*, b* values, the difference in the lightness (∆L*) and chroma coordinates (∆a* and ∆b*), hue angle (h), saturation (C*), and total color difference (∆E) were calculated using the following formula.
∆L* = L*_t_ − L*_c_h = *arctg*(b*/a*)∆a* = a*_t_ − a*_c_C* = (a*^2^ + b*^2^)^1/2^∆b* = b*_t_ − b*_c_∆E = (∆L*^2^ + ∆a*^2^ + ∆b*^2^)
where, L*_t_, a*_t_, b*_t_ are L*, a*, and b* of the heated specimens; L*_c_, a*_c_, b*_c_ are L*, a*, and b* of the control specimens, respectively.

**Figure 7:**
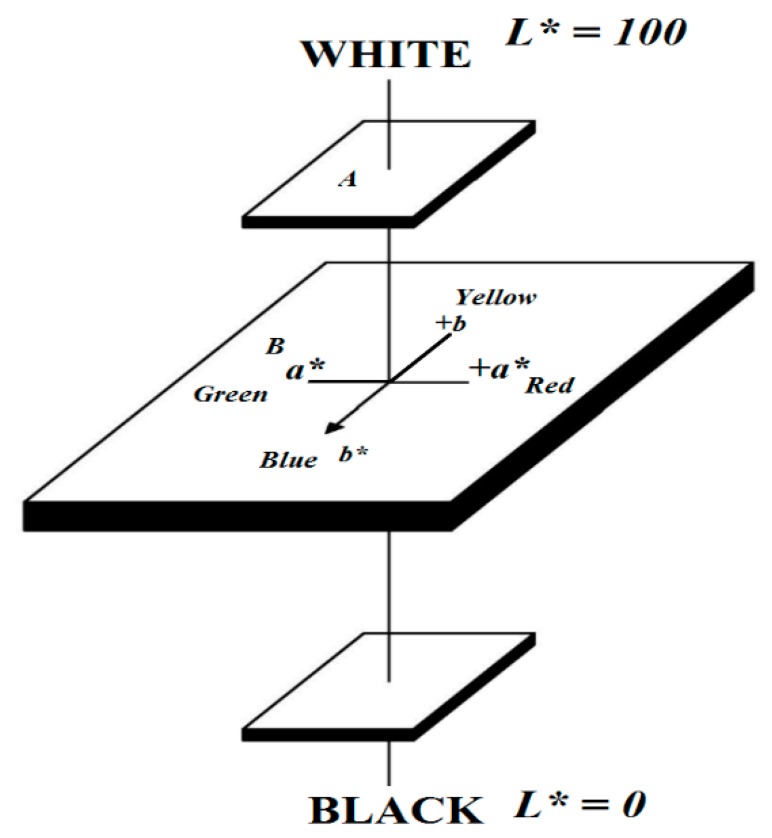
Color space defined by the L*, a*, b* axes. In addition, the deduced color parameter hue angle h is indicated (A: Color of occupation; B: Color of specimens; A’: Color of occupation at equal lighting as color of specimens).

On the hue circle, h = 0° denotes redness and h = 90° denotes yellowness, as illustrated in Figure 7. Saturation C*, corresponding to the distance between the color and the center of the chromaticity plane, is a measure of color intensity. The measurements of color were determined according to DIN 5033. The measurements are average values with standard deviations [20].

### 2.8. Data Analysis

One-way analysis of variance (one-way ANOVA) was performed to analyze the significant differences of all parameters used in this study. All results were computed using IBM Statistical Package for the Social Sciences (SPSS) software (IBM Inc., Armonk, NY, USA).

## 3. Results and Discussion

Table 2 displays test results of the specimens. The average density of the control samples was 0.46 g/cm^3^. Overall density levels of the samples increased substantially with increasing press temperature. The increase was the highest in the case of the specimens having density of 0.93 g/cm^3^ compressed at a temperature of 180 °C. The moisture contents for the densified samples were 6–8%. 

### 3.1. Effect of the Temperature Level on Mechanical and Physical Properties

The analysis of variance related to the effectiveness of the temperature level on densification enhanced overall mechanical properties of the samples, as displayed in Table 3.

The densification process was found to be effective on the density, adhesive bondline shear strength, surface roughness perpendicular and parallel to grain orientation, lightness, hardness in radial and tangential directions of eastern redcedar specimens (*p* < 0.05). Table 4 displays the homogeneity test results related to the temperature levels that created a difference according to the values determined in this work.

In this study, five groups of heat treatment 100 °C, 120 °C, 150 °C, 180 °C, and control groups were used to evaluate the of effect of temperature on physical and mechanical properties of the samples. All properties of the samples considered in this study were significantly changed compared with those of the control groups (*p* < 0.05) as a result of heat treatment. By comparing the density of heat treatment groups with control group, a significant increase was observed at temperature levels 100 °C, 120 °C, 150 °C, 180 °C, however a remarkable similarity was found between the heat treatment groups after exposure to a temperature of 120 °C. A substantial decrease in all specimen properties was also observed in all heat treatment groups. Color changes of the samples significantly decreased for those exposed to 100°C (*p* < 0.05), while the overall values of hardness in radial and tangential directions after heat treatment significantly increased for the group exposed to a temperature of 100 °C. On the other hand, hardness in radial and tangential directions started to decrease with exposure to temperature groups 150 °C and 120 °C. Table 4 displays comparative test results. 

#### 3.1.1. Evaluation of Density

**Figure 8:**
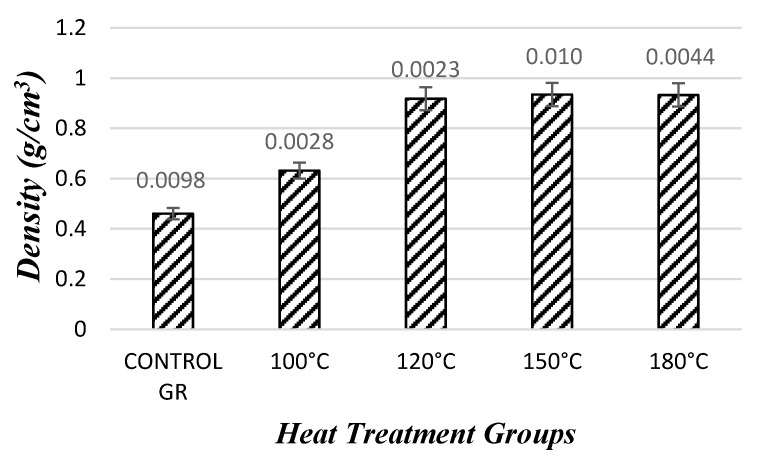
Density values of the samples after the densification process.

Since the samples reached a certain threshold point of densification, no significant difference in their density values was observed when they were compressed at temperature levels of 120 °C, 150 °C, and 180 °C. However, statistical analysis resulted in significant difference for the values between control samples and those compressed at a temperature of 100 °C and the others, as illustrated in Figure 8. 

After the densification process, the density of the specimens increased from 0.46 g/cm^3^ to 0.93 g/cm^3^ at all temperature levels, resulting in a 101% density increase. The control specimens and densified specimens appeared in separate homogeneity groups. 

#### 3.1.2. Evaluation of Adhesive Bondline Shear Strength Test

The control sample had an average adhesive bondline shear strength of 36.60 MPa, which was the highest value of the samples. Once samples were compressed under the influence of temperature levels of 100 °C 120 °C, 150 °C, and 180 °C, they had adhesive bondline shear strength values of 15.13 MPa, 11.2 MPa, 13.42 MPa, and 16.00 MPa, respectively. It appears that compression and heating the samples adversely influenced their adhesive bondline shear strength. It is a fact that a smoother surface will create less interaction between two samples, resulting in lower adhesive bondline shear strength characteristics.

In a previous study, adhesive bondline shear strength of four Japanese species (namely cedar-sugi, cypress-hinoki, arborvitac-hiba, and larch-karamatsu) had lower shear strength properties when they were sanded with finer sand paper as compared to those of sanded with coarser sand paper [21]. 

**Figure 9:**
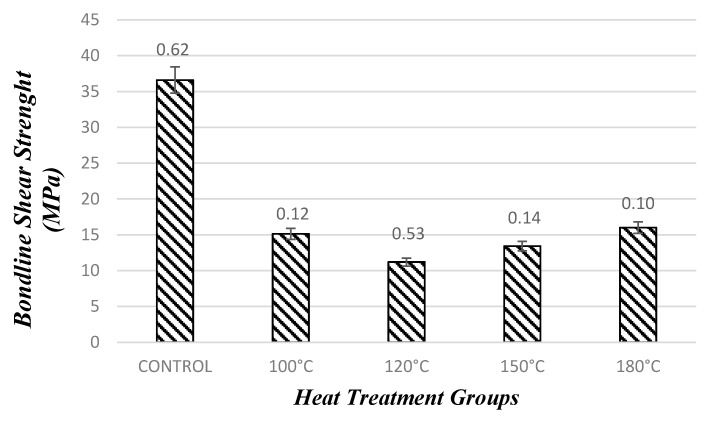
Adhesive bondline shear strength values of the samples after densification process.

In this study, compression and heat also created smoother surface quality of the samples and adversely effected the development of a strong glue line between two pieces. As temperature was increased from 100 °C to 120 °C and 150 °C, the reduction in bonding strength of the specimens was determined. They were slightly reduced, as can be observed in Figure 9.

It is known that physical and chemical changes in the wood take place during the application of compression with heat. This could possibly be the main reason for such findings. Additionally, the combined effect of heat and compression affects and promotes an inactivated wood surface, resulting in adhesion problems.

#### 3.1.3. Evaluation of Surface Roughness Tests

Roughness values taken from the surface of specimens in orientations both across and along the grain improved due to compression. Control species had an average value of R_a_ perpendicular to the grain of 8 μm, while this value reduced to 4.12 μm, 3.64 μm, 3.56 μm, and 1.22 μm for those samples compressed at temperatures 100 °C, 120 °C, 150 °C, and 180 °C, respectively, Control species had an average value of R_a_ parallel to the grain of 3.54 μm, while this value reduced to 1.28 μm, 1.38 μm, 1.16 μm, and 1.10 μm for samples compressed at temperatures 100 °C, 120 °C, 150 °C, and 180 °C, respectively.

**Figure 10:**
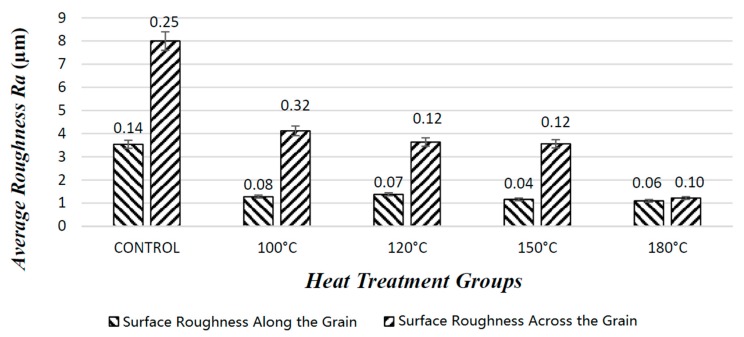
Surface roughness values of the samples after densification process.

In a previous work, a 40% reduction in the surface roughness of poplar veneer was observed when they were compressed in a press [13], and roughness values resulted in significant differences between the measurements taken from the surface of the samples parallel and perpendicular grain orientation. In all cases, samples compressed at a temperature of 180 °C did not show any differentiation. It appears as if compression at a temperature of 180 °C made the surface layer of the samples substantially soft and plasticized. Figure 10 shows roughness values taken from the surface after the densification process. 

Overall, the smoothness of the samples improved with increasing treatment temperature. Significant differences between surface roughness values control and compressed samples as well as values within compressed samples were found based on statistical analysis. 

#### 3.1.4. Evaluation of Lightness Test

**Figure 11:**
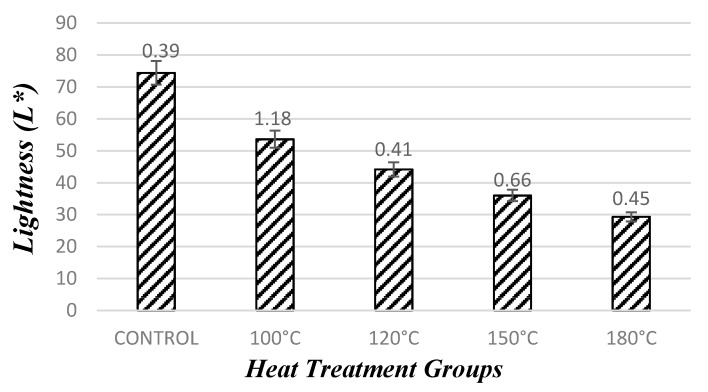
Color values of the samples after densification process.

Densification with heat treatment significantly changed the color values of the samples. The lightness (L*) value was measured as higher (74.39) for control samples. After densification with heat treatment, the L* value of samples significantly decreased depending on the increase of temperature levels. When compared to control samples, the L* value decreased to 53.60, 44.17, 35.99, and 29.32 for samples compressed at temperature levels of 100 °C 120 °C, 150 °C, and 180 °C, respectively, as depicted in Figure 11. 

In previous studies, it has been expressed that the color of the wood darkened more with the increasing temperature and duration [20,22,23,24,25]. Overall lightness of the samples decreased with treatment temperature. Significant differences between lightness values of control and compressed samples as well as values within compressed samples were found based on statistical analysis. 

#### 3.1.5. Evaluation of Hardness Tests

**Figure 12:**
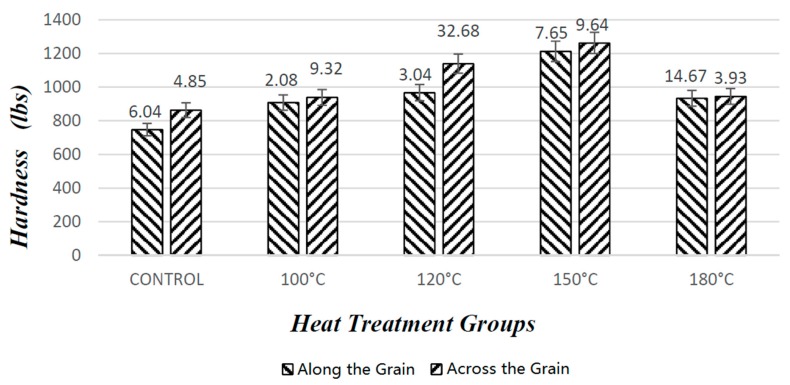
Hardness values of the samples after the densification process.

Figure 12 shows hardness values of the specimens in both grain orientations. The highest value was 1262 lbs across the grain orientation of the specimens exposed to a temperature of 150 °C. As press temperature increased, overall hardness characteristics of the samples also gradually increased. 

Including control samples, all of the specimens had higher hardness taken from perpendicular to the grain orientation than that taken parallel to the grain orientation, which is the expected result due to the anatomical structure of wood material. It seems that 150 °C temperature was the threshold point for hardness values of the samples. Beyond this point, when samples were compressed at a temperature of 180 °C, their hardness values reduced 23% parallel to the grain orientation and 25% perpendicular to the grain orientation, as can be observed in Figure 12. It is a known fact that high temperature close to 200 °C creates a certain amount of degradation of the chemical structure, and this is reflected in the adverse influence on mechanical properties.

## 4. Conclusions

This study investigated the influence of thermomechanical densification on surface quality, adhesive bondline shear strength, hardness, and color changes of densified eastern redcedar. Based on the findings in this work, overall surface roughness characteristics of samples exposed to different temperatures were enhanced as a result of densification. This positive effect was more pronounced in the care of the sample exposed to a temperature of 120 °C. Data found in this work are useful for more efficient use of wood as a value-added product from eastern redcedar which is a lesser utilized species. In further studies, some of the other mechanical properties such as bending and compression strength in addition to dimensional stability of the samples would be interesting to investigate to have a better understanding of the behavior of densified eastern redcedar.

## Figures and Tables

**Table 1:** Dimensions of the samples densified using heat and pressure.

Applied Tests	Size of Specimens Before Densification (mm)	Size of Specimens After Densification (mm)	Number of Specimens	
			Control	Densified
Density	50 × 40 × 20	50 × 40 × 14	5	20
Sheer Strength	90 × 40 × 20	90 × 40 × 14	5	20
Surface Roughness Parallel to the Grain	50 × 40 × 20	50 × 40 × 14	5	20
Surface Roughness Perpendicular to the Grain	50 × 40 × 20	50 × 40 × 14	5	20
Janka Hardness Strength Radial D.	50 × 40 × 20	50 × 40 × 14	5	20
Janka Hardness Strength Tangential D.	50 × 40 × 20	50 × 40 × 14	5	20
Color Differences	50 × 40 × 20	50 × 40 × 14	5	20

**Table 2:** Average physical and mechanical properties of densified eastern redcedar at a pressure of 6.08 MPa and different temperature levels.

Sample Types	Statistical Value	Density (g/cm^3^)	Bondline Shear Strength (MPa)	Surface Roughness (Ra)		Lightness L*	Hardness	
				Across the Grain (μm)	Along the Grain (μm)		Radial (lbs)	Tangential (lbs)
Control	Mean	0.4604	36.60	8.00	3.54	74.39	747.20	863.20
	Standard Dev.	0.0219	1.40	0.56	0.32	0.88	13.51	10.84
Densified at 100 °C	Mean	0.6316	15.13	4.12	1.28	53.60	908.20	939.00
	Standard Dev.	0.0064	0.28	0.72	0.17	2.64	4.65	20.84
Densified at 120 °C	Mean	0.9180	11.20	3.64	1.38	44.17	966.60	1138.80
	Standard Dev.	0.0051	1.18	0.27	0.16	0.92	6.80	73.08
Densified at 150 °C	Mean	0.9340	13.42	3.56	1.16	35.99	1262.20	1212.40
	Standard Dev.	0.0225	0.32	0.28	0.08	1.48	21.56	39.48
Densified at 180 °C	Mean	0.9320	16.00	1.22	1.10	29.32	933.80	945.00
	Standard Dev.	0.0099	0.23	0.23	0.14	1.02	32.82	8.80

**Table 3:** Analysis of variance related to the effect of the temperature level on density.

Applied Tests	Statistical Values	Sum of Squares	Level of Significance (*p* ≤ 0.05)
Density	Between Groups	95,1473.36	0.000
	Within Groups	4638.80	
	Total	96,112.16	
Adhesive Bondline Shear Strength	Between Groups	2121.47	0.000
	Within Groups	14.53	
	Total	2136.01	
Surface Roughness Across to Grain	Between Groups	120.03	0.001
	Within Groups	4.24	
	Total	124.27	
Surface Roughness Along to Grain	Between Groups	21.57	0.000
	Within Groups	0.76	
	Total	22.33	
Lightness	Between Groups	6172.75	0.000
	Within Groups	47.47	
	Total	6220.23	
Hardness in Radial Direction	Between Groups	561,002.96	0.000
	Within Groups	11,546.80	
	Total	572,549.76	
Hardness in Tangential Direction	Between Groups	545,846.64	0.001
	Within Groups	13,890.40	
	Total	559,737.04	

**Table 4:** Comparative test results for the effect of densification on various properties of the samples at different temperatures for homogeneity groups.

Physical and Mechanical Tests	Densification Temperature (°C)	H.G. * A	H.G. * B	H.G. * C	H.G. * D	H.G. * E
Density	Control Group			0.460		
	100 °C		0.631			
	120 °C	0.918				
	150 °C	0.934				
	180 °C	0.932				
Bondline Shear Strength	Control Group	36.60				
	100 °C		15.13			
	120 °C				11.20	
	150 °C			13.42		
	180 °C		16.00			
Surface Roughness Across the Grain Orientation	Control Group	8.00				
	100 °C		4.12			
	120 °C		3.64			
	150 °C		3.56			
	180 °C			1.22		
Surface-Roughness Along the Grain Orientation	Control Group	3.54				
	100 °C		1.28			
	120 °C		1.16			
	150 °C		1.38			
	180 °C		1.10			
Lightness	Control Group	73.39				
	100 °C		53.60			
	120 °C			44.17		
	150 °C				35.99	
	180 °C					29.32
Hardness in Radial Direction	Control Group				747.20	
	100 °C			908.20		
	120 °C		966.60			
	150 °C	1212.40				
	180 °C			933.80		
Hardness in Tangential Direction	Control Group				863.20	
	100 °C			939.00		
	120 °C		1139.20			
	150 °C	1262.20				
	180 °C			945.00		

* Homogeneity groups.
